# Clinical Evaluation of a Real-Time Wearable System for Monitoring In-Hospital Ambulatory Patients With COVID-19: Retrospective Data Study

**DOI:** 10.2196/81304

**Published:** 2026-06-22

**Authors:** Sarah Vollam, Cristian Roman, Mauro Santos, Marco Pimentel, Oliver Redfern, Lionel Tarassenko, Peter Watkinson

**Affiliations:** 1 Nuffield Department of Clinical Neurosciences University of Oxford Oxford United Kingdom; 2 NIHR Oxford Biomedical Research Centre Oxford United Kingdom; 3 Institute of Biomedical Engineering Department of Engineering Science University of Oxford Oxford United Kingdom; 4 Oxford Critical Care John Radcliffe Hospital Oxford United Kingdom

**Keywords:** vital signs, ambulatory monitoring, patient safety, early warning score, nurse workload, wearable devices

## Abstract

**Background:**

Many studies have evaluated the use of wearable monitoring systems to improve patient safety in hospital. Although some have demonstrated effects on intensive care admissions, there remains little evidence of impact on patient outcomes such as mortality, hospital length of stay, and time to antibiotic administration. Very few studies have focused on how wearable monitoring systems are used in clinical practice, including how the rate of manual vital sign measurements (MVSMs) is affected.

**Objective:**

Our primary aim was to describe the physiological pattern of vital signs in hospitalized patients treated for COVID-19 outside of critical care. We also report an exploratory post hoc analysis of the impact of displaying wearable monitoring system data on the frequency of intermittent MVSMs.

**Methods:**

We conducted a retrospective study during the COVID-19 pandemic following deployment of a wearable monitoring system that continuously displayed heart rate, respiratory rate, and oxygen saturation levels. We included patients treated for COVID-19 in 3 isolation wards in a large UK hospital. Wearable monitoring system data were displayed on a dashboard in the center of each ward. We analyzed the patterns of vital signs in patients monitored using the wearable monitoring system. We compared the time to next observation (led by nursing staff) for routinely collected MVSMs between periods when patients were continuously monitored and those when they were not. In exploratory post hoc analysis, we tested whether the difference varied between stable (early warning score [EWS] above the escalation threshold) and unstable patients.

**Results:**

Patients (N=144) had continuous vital signs above the EWS threshold for escalation for 32.7% (2133/6528) of time monitored. The unadjusted median time between MVSMs for continuously monitored periods was 39 minutes (95% CI 29-49; *P<*.001) longer than for unmonitored periods. When adjusted for EWS category and participant-level clustering, the effect was attenuated but remained significant (14.6 minutes; *P*<.001). In exploratory post hoc analysis, we found that increases were larger during stable observation periods (51 minutes, 95% CI 39-62; *P<*.001) than during unstable periods (16 minutes, 95% CI 8-24; *P*<.001). However, adjusted analyses did not support a significant difference between stable and unstable periods.

**Conclusions:**

Patients in this study were at elevated risk of deterioration, spending a third of monitored time at or above the escalation threshold. We found that, by offering additional vital sign data between manual measurements, the time between routine MVSMs increased, which may reflect changes in nursing task prioritization. Although patient safety outcomes were not directly measured, we found no indication that reducing observation frequency adversely affected patient safety.

## Introduction

### Background

Among patients in the United Kingdom hospitalized with COVID-19 during the pandemic, 42% required ventilatory support or high-dependency or critical care or died in hospital [1]. Therefore, patients diagnosed with COVID-19 managed in hospital wards required close monitoring of vital signs to ensure that deterioration was identified promptly and care was escalated appropriately. In usual care, patients at high risk of deterioration are placed in high-visibility areas of the ward so that nurses can observe them closely. However, due to the elevated risk of COVID-19 transmission both to other patients and staff, patients were often cared for in single isolation rooms or closed cohort bays [2]. Isolation, plus the time required by staff to don personal protective equipment (PPE), posed challenges to the close observation required to ensure the safety of these patients who are acutely ill.

Continuous monitoring of vital signs using wearable devices has long been recognized as having the potential to detect deterioration earlier than intermittent measurements [3-5]. Early in the pandemic, it was recognized that patients diagnosed with COVID-19 had elevated respiratory rates and rapidly worsening oxygen saturation levels that required high levels of supplemental oxygen [6]. As a result, the use of wearable vital sign monitoring escalated, with devices commonly used to allow low-risk patients to be managed at home [7].

At the start of the COVID-19 pandemic, we were close to completing development of a wearable system for the continuous monitoring of heart rate, respiratory rate, and oxygen saturation in hospital wards as part of a research project. Multiple phases of development had been completed that assessed the wearability, accuracy, and reliability of the wearable devices and system [8-10]. Given the concerns about maintaining patient and staff safety in wards with patients with COVID-19, it was clear that this system had the potential to support local clinical care in wards receiving patients with COVID-19. Implementation was therefore accelerated, and the system was deployed in a ward in late March 2020, the day on which the United Kingdom first entered lockdown, with the aim of supporting nurses in managing these high-risk patients.

Although wearable monitoring is increasingly being used in ward-based care internationally to support patient safety [3], little is known about the mechanism of impact [5]. Studies to date have focused on improving patient safety–related outcomes (such as in-hospital mortality and length of stay) [3], whereas studies of staff and patient views of wearable monitoring system (WMS) use have focused on safety and reassurance [11-13]. The presence of WMSs alone cannot improve patient outcomes, and the impact of WMSs on nurse behaviors related to vital sign monitoring is not well understood [5].

### Aim

The primary aim of this retrospective data study was to describe the physiological pattern of vital signs over the course of SARS-CoV-2 infection for hospitalized patients outside of critical care. In this paper, we also report a post hoc analysis exploring the impact of displaying data from the WMS on the timing of nurses recording intermittent manual vital sign measurements (MVSMs).

## Methods

The system was implemented into usual care as part of a local service evaluation (Datix: 5973). Following deployment of the system, we sought approval to conduct a retrospective data study linking continuous vital sign data from wearable monitoring with early warning score (EWS) values from the electronic patient record (EPR) and limited outcome data, which were then pseudonymized and extracted from the hospital’s EPR (Cerner Millennium [14]).

### Setting

The service evaluation was undertaken in an isolation ward with 19 side rooms and two 24-bed medical wards where patients with COVID-19 were cohorted in open bays within a large tertiary referral hospital in the United Kingdom.

### Participants

We included all patients diagnosed with COVID-19 (confirmed via laboratory polymerase chain reaction test) and remotely monitored at any point during their stay in 1 of the 3 wards where we deployed the wearable system between March 23, 2020, and February 28, 2021.

### Vital Signs Monitoring

#### Intermittent EWS Measurements

During the service evaluation period, a locally developed EWS was used [15,16]. According to local policy, based on national guidance at the time (prior to implementation of the National Early Warning Score 2), patients with an EWS of 0, 1, or 2 required observations every 4 hours. For a score of 3 or more, the frequency of observations was increased to hourly measurements. Vital sign measurements taken intermittently by nurses were obtained using their usual ward equipment and recorded in an electronic device that transferred data to the EPR for each patient [17].

#### Wearable Monitoring System

We published the technological aspects of the system previously [18]. The WMS consists of a chest-worn patch monitoring heart rate and respiratory rate (VitalPatch; VitalConnect) and a wrist-worn pulse oximeter measuring pulse rate and peripheral oxygen saturation (SpO_2_; Nonin WristOx2 3150). Both devices were connected via Bluetooth Low Energy to a bedside Android Samsung Galaxy Tab A (2016). Partial EWSs were calculated every 5 minutes for the continuous wearable data by adding the individual scores for heart rate, respiratory rate, and SpO_2_ obtained by computing the 5-minute medians for each of these variables.

The WMS was developed as part of a research project following extensive wearability, functionality, and usability testing [3,9,18] leading to selection of the most reliable, wearable, and accurate devices. Selection of the pulse oximeter device was supported by reliability testing, including hypoxia testing, which identified extensive variability in accuracy between several devices. The device finally selected for the WMS was found to underestimate SpO_2_ values by up to 2% [9]. However, it was assessed as the most reliable device as it under- rather than overestimated oxygen saturation (deemed to be safer in clinical practice) and was the most reliable in terms of connectivity and usability. This potential underestimation of oxygen saturation was conveyed to staff during training in the use of the system.

Table S1 in Multimedia Appendix 1 shows the sources of each vital sign recorded by the WMS and the intermittent nurse observations. The levels of agreement between these 2 sources of vital sign data, presented in Figures S1-S4 in Multimedia Appendix 1, reflect measurements obtained under routine ward conditions, including patient movement and intermittent removal or adjustment of oxygen interfaces at the time of nurse observation, rather than a controlled validation setting.

#### Staff Interface

Continuous vital sign data were transmitted in real time from the Android tablet to an in-hospital server installation via Wi-Fi. A user interface displayed real-time and time-stamped 5-minute median estimates of heart rate, respiratory rate, and SpO_2_ (as well as the 1-lead electrocardiogram and photoplethysmogram waveforms) and corresponding partial EWSs alongside intermittent vital sign measurements recorded by the nursing staff and the corresponding overall EWS. The interface used a “traffic light” scheme in displaying these data, with individual cards highlighted in green (EWS of 0), amber (EWS of 1-2), and red (EWS of ≥3) according to the calculated partial EWS from wearable monitoring. This was displayed on dedicated desktop screens at the central nurses’ station in each ward. No audible or visual alerts were implemented. The user interface was also viewable on other devices (using log-in credentials) by research and clinical staff. In total, 20 sets of wearable monitoring equipment were available to staff in the 3 wards, and monitoring use was based on the clinical judgment of the nursing staff.

### Data Extraction and Management

Pseudonymized data were retrospectively extracted from the local National Health Service (NHS) hospital data warehouse by hospital employees and associated with the wearable data via a link table (mapping local record numbers to system identifiers) after applying the NHS digital data opt-out service. All data were stored on a secure hospital server for analysis and only accessible within the hospital network.

Data extraction was limited to the following variables:

- Patient demographics (age and sex)
- Ward admission and discharge time stamps
- Intermittently measured vital sign values, corresponding EWSs, and time stamps
- Outcome data, including in-hospital death, admission to the intensive care unit (ICU), and cardiac arrest call

Extracted data were limited to the duration of hospital admission. There was no follow-up, and study duration ended at death or hospital discharge.

All patients had periods without continuous monitoring data before and/or after being on the WMS. These data were split into 2 datasets: periods A (periods without wearable monitoring data—“unmonitored periods”) and B (periods during which wearable monitoring data were recorded—“monitored periods”). Therefore, the 2 datasets were generated from the same cohort of patients. Given the retrospective design, it was not possible to access intermittent vital sign data from a nonmonitored cohort of patients.

Patients with no monitoring data were not included in the analysis. Once a patient was considered to have been monitored using wearable devices, data availability was calculated as the percentage of at least one vital sign value being received within a 5-minute interval between the first and last wearable data point received.

### Analysis

#### Time to Next Observation

**Figure 1:**
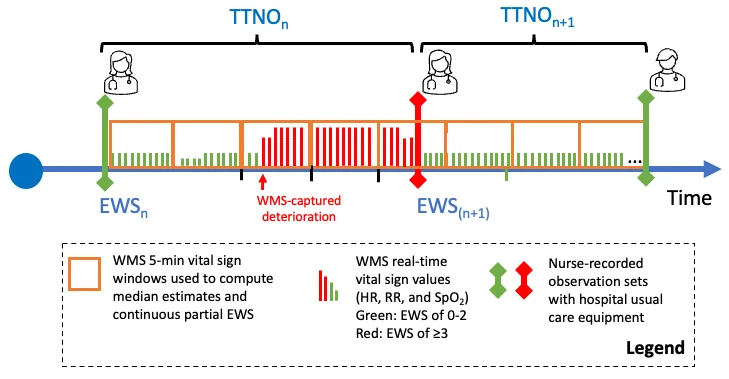
Visualization of time to next observation (TTNO) and wearable data capture. EWS: early warning score; HR: heart rate; RR: respiratory rate; SpO2: peripheral oxygen saturation; WMS: wearable monitoring system.

The time to next observation (TTNO) was defined as the time interval in minutes between 2 consecutive nurse-led recorded observation sets (EWS_n_ and EWS_n+1_; Figure 1). We compared the TTNO between periods A (unmonitored periods) and B (monitored periods) and between subgroups of “stable” (EWS of 0-2) and “unstable” (EWS of ≥3) periods, reporting the median and 95% CIs.

#### Distribution of TTNO Values

Distribution of TTNO values was split into quartiles and compared between periods A (unmonitored periods) and B (monitored periods). To explore the potential safety impact of monitoring, we compared the change in EWS between consecutive nurse observations when nurse observation was much later than expected (within the highest quartile [fourth] of the TTNO distribution) for unmonitored periods vs monitored periods.

Only complete sets of intermittent vital sign measurements from nurse observations (heart rate, respiratory rate, SpO_2_, blood pressure, and temperature) were included in the analysis. We excluded all EWS_n+1_ sets with a TTNO of less than 10 minutes as we assessed that the nurse was unlikely to have left the isolation room between 2 sets of measurements so close in time. We excluded EWS_n+1_ sets with a TTNO of 900 minutes or more as this indicated that the patient had left the ward for a prolonged period (ie, transferred from and subsequently readmitted to the ward).

#### Statistical Analysis

Statistical analysis was conducted using Python (Python Software Foundation) and R software (R Foundation for Statistical Computing). Medians, IQRs, and normalized histograms (Seaborn version 12.1 [19]) were used to compare the TTNOs between the unmonitored and monitored periods. Bootstrap with replacement (n=5000) was used to compute 95% CIs. The Mann-Whitney *U* test was used to determine significance (*P*<.05) between skewed distributions. The *z* test was used to determine significance (*P*<.05) between normal distributions (confirmed using the Shapiro test).

Using the GAMLSS library (version 5.4.3) [20], the following mixed effects model was applied to test for the effect of the monitoring period on the TTNO (in Wilkinson notation): TTNO_n_ ~ EWS_unstable_n_ + monitoring_period_n_ + (1 participant), where TTNO_n_ is the TTNO from the current observation set *n*, EWS_unstable_n_ is a binary variable with a value of 1 if the EWS for the current set of observations *n* (which drives the TTNO) was equal to or above 3 and 0 otherwise, and monitoring_period_n_ is a binary variable with a value of 1 if the patients were monitored using the wearables at the time of the set of observations *n* (both variables modeled as fixed effects). Random effects were considered for each participant (1|participant), indicating that the model includes a random intercept for each participant. We assumed a normal distribution for the random effects and error term. Sensitivity analyses using gamma and log-normal mixed models were performed to assess the impact of skewness. An α value of .05 was used to assess the significance of the effect of wearable monitoring (monitoring_period_n_). As a robustness check, the model was also re-estimated treating EWS as a continuous variable rather than a binary variable. An interaction between clinical stability and monitoring period was also explored as an additional analysis.

WMS-recorded EWS trajectories were summarized relative to normalized hospital length of stay (0%-100%) to mitigate confounding introduced by variable lengths of stay and to align patients by comparable phases of hospitalization or relative to normalized wearable monitoring time to align trajectories by comparable phases of monitoring regardless of monitoring duration. Trajectories indexed to relative monitoring time should be interpreted with caution as varying monitoring durations may introduce survivorship and compositional biases at later time points, where fewer patients contribute to the estimates. At each relative percentage point, the mean of the maximum EWS values from all contributing patients was calculated to generate group-level trajectories. The 95% CIs were estimated using nonparametric bootstrap resampling. WMS-recorded EWS trajectories were also compared between patient subgroups defined as wave 1 (patients monitored between March 2020 and August 2020) and wave 2 (patients monitored between September 2020 and February 2021) [21]. Analyses were descriptive and intended to characterize patterns of EWS trajectories across patient groups.

### Ethical Considerations

As this was a retrospective data study that did not use any identifiable data, Health Research Authority approval was sought and granted (12/HRA/1234), and the study was registered (ISRCTN 85624923).

## Results

### Overview

**Figure 2:**
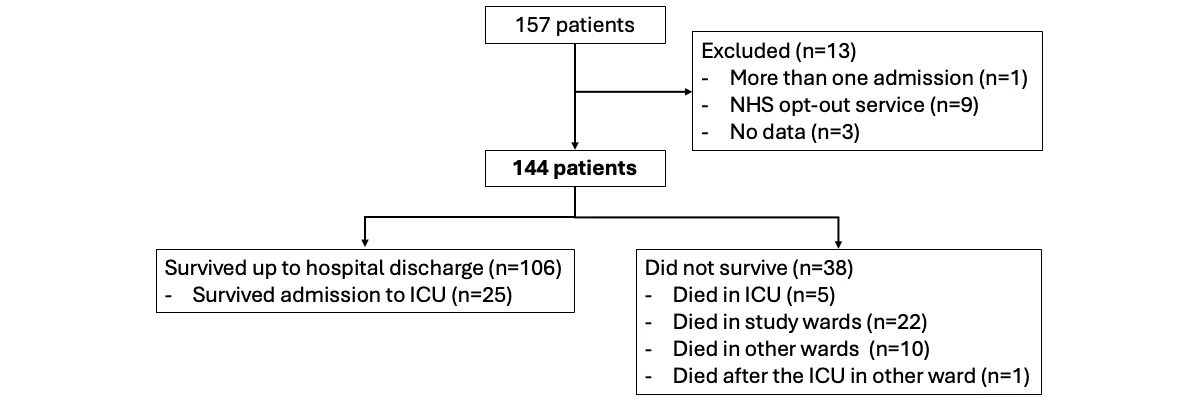
Patient cohort flow diagram. ICU: intensive care unit; NHS: National Health Service.

A total of 157 unique patients were monitored using the WMS between March 23, 2020, and February 28, 2021, with data on 144 (91.7%) patients available after exclusions were applied (Figure 2). There were no missing outcome data from included patients. The median age was 62.5 (IQR 47-77) years, and 29.9% (43/144) of the patients were female. The age distributions of patients admitted during waves 1 and 2 are shown in Figure S5 in Multimedia Appendix 1. Of the 144 patients included in the analysis, 97 (67.4%) were admitted to side rooms in an isolation ward, and 47 (32.6%) were admitted to cohorted bays in 2 medical wards.

### Trajectory of Illness and Pattern of Vital Signs During SARS-CoV-2 Infection

Of the 144 patients available for analysis, 38 (26.4%) died during their hospital admission, 31 (21.5%) were admitted to ICU, and none had a cardiac arrest call. The duration of hospital stay ranged from 0.7 to 141.9 (median 8.9, IQR 5.6-19.8) days. The wearable monitoring period ranged from 0.15 to 440.2 (median 35.5, IQR 9.23-97.02) hours. Once a patient was placed on wearable monitoring, the median data availability was 88.5% (IQR 71.95%-98%). A total of 11,634 intermittent EWSs were recorded. During wearable monitoring, patients had continuous vital sign values that exceeded the EWS escalation threshold for 32.7% (2133/6528) of the total hours. The maximum intermittent EWS recorded was 17. The maximum continuous/partial EWS recorded was 9. The median maximum continuous/partial EWS for the duration of waves 1 and 2 was 3 (IQR 2-4) and 2 (IQR 1-4), respectively. Additional graphs showing the patterns of vital signs based on patient outcome and pandemic wave can be found in Multimedia Appendix 2.

### TTNO Results

**Figure 3:**
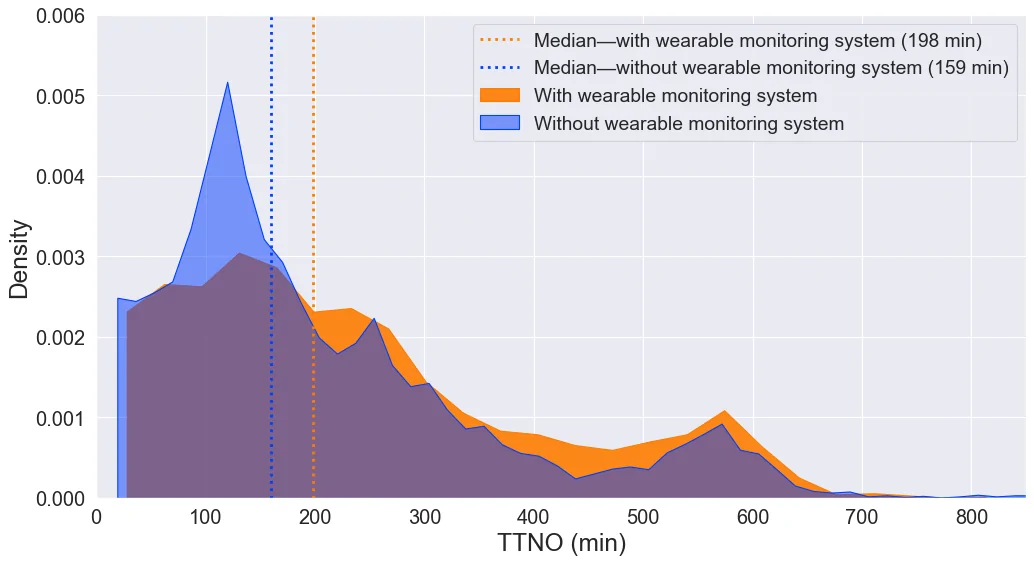
Normalized histogram of time to next observation (TTNO) values for all patients with and without wearable monitoring.

For all data, the median increase in TTNO for monitored periods was 39 minutes (95% CI 29-49; *P*<.001; Table 1) longer than unmonitored periods. Figure 3 compares the distribution of TTNO values for these 2 periods, with the median values reported in Table 1 for the EWSs of all patients. Figure 3 shows that the 120-minute peak in TTNO for unmonitored periods was not present in monitored periods.

For stable periods (EWS of 0-2), the increase in median TTNO during monitored periods was 51 minutes (95% CI 39-62; *P*<.001) compared with unmonitored periods (Table 2). For unstable periods (EWS of ≥3), the increase in median TTNO during monitored periods was 16 minutes (95% CI 8-24; *P*<.001; Table 2).

**Table 1:** Comparison of median times to next observation (TTNOs) with and without wearable monitoring for all the early warning scores (0-17) of all patients.

		TTNO^a^ (min), median (IQR; 95% CI)	Difference (monitored – unmonitored; min; 95% CI)		*P* value	
**EWS of 0-17**				+39 (29-49)		<.001
	Without wearables (n=8866 TTNOs)	159 (101-275; 155-163)				
	With wearables (n=1963 TTNOs)	198 (109-326. 190-208)				

^a^Reported sample sizes exclude TTNO values of less than 10 minutes or of 900 minutes or more, as described in the Methods section.

**Table 2:** Comparison of median time to next observation (TTNO) with and without wearable monitoring for stable periods (early warning score [EWS] of 0-2) and unstable periods (EWS of ≥3).

		TTNO^a^ (min), median (IQR; 95% CI)	Difference (monitored – unmonitored; min; 95% CI)	*P* value
**EWS of 0-2**			+51 (39-62)	<.001
	Without wearables (n=6043 TTNOs)	187 (116-310; 182-192)		
	With wearables (n=1240 TTNOs)	238 (146-386; 228-248)		
**EWS of ≥3**			+16 (8-24)	<.001
	Without wearables (n=2823)	123 (74-193; 120-126)		
	With wearables (n=723)	139 (76-239; 131-146)		

^a^Reported sample sizes exclude TTNO values of less than 10 minutes or of 900 minutes or more, as described in the Methods section.

### Mixed Effects Model

The mixed effects model results for all patients (Table 3) show that both the use of wearable monitoring and unstable EWS periods were statistically significant (*P*<.001 for both terms) in association with TTNO. When adjusting for EWS category and participant-level clustering, the effect was attenuated but remained significant (14.6 minutes; *P*<.001). Re-estimating the model with EWS treated as a continuous variable showed similar results (*P*<.001 for both terms). Sensitivity analyses using gamma and log-normal mixed models showed consistent results in terms of direction and statistical significance. There was no evidence of interaction between clinical stability and monitoring period (*P*=.35), indicating that the effect of wearable monitoring on TTNO did not differ significantly between stable and unstable observations.

**Table 3:** Impact of early warning score (EWS) and monitoring period on the time to next observation for all EWSs (current observations)a.

	Fixed-effect coefficient (SE)	*t* test	*P* value
Intercept	239.043 (1.822)	131.234	<.001
Unstable EWS (≥3)	−83.129 (2.995)	−27.757	<.001
Wearable monitoring period	14.634 (3.648)	4.011	<.001

^a^Coefficients represent fixed effects from the GAMLSS model. *t* values are calculated as the ratio of the coefficient to its SE, and *P* values are based on asymptotic inference. Residual for the fitted model were 10707.9.

### Impact of Delayed Vital Signs Measurements

**Figure 4:**
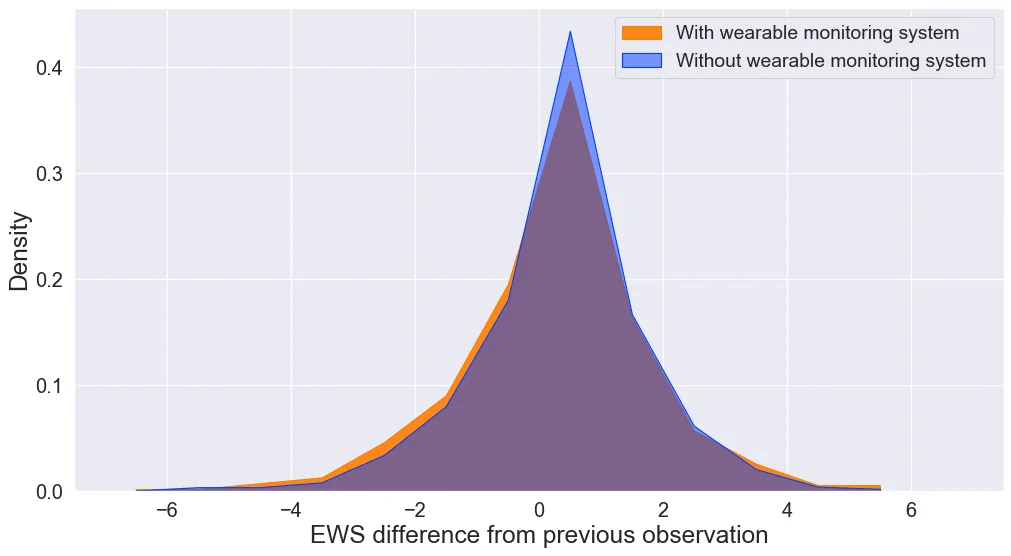
Normalized histogram of early warning score (EWS) differences between the current and previous set of observations for times to next observation within the fourth quartile.

Figure 4 compares normalized score differences in EWS in the fourth quartile of TTNO for periods A (unmonitored periods: 275-898 minutes) and B (monitored periods: 326-830 minutes), demonstrating little difference between them (*P*=.92).

## Discussion

### Principal Findings

#### Overview

In this retrospective data analysis study of a large dataset of 11,634 vital signs measurements, we mapped the trajectory of vital signs during SARS-CoV-2 infection. We found that this cohort was acutely unwell, with almost a third of the continuous monitoring duration at or above the threshold for escalation and a very high peak of nurse-recorded EWSs at 17. This is reflected in the outcomes for this patient group, with 26.4% (38/144) of patients dying in hospital and 21.5% (31/144) admitted to intensive care.

#### TTNO Measurement

Our post hoc analysis suggests that the availability of continuous patient monitoring data using clinical-grade wearables enabled a change in nurse behavior. Nursing staff increased the time they took before they returned to a patient to make the next set of observations by 39 minutes (*P*<.001) in unadjusted analysis. The effect was attenuated but remained significant (14.6 minutes; *P*<.001) when adjusted for EWS category and participant-level clustering. This would indicate that the nurses trusted the vital sign data acquired by the wearable system, which enabled them to monitor patients remotely using the live data and 5-minute median summaries of heart rate, respiratory rate, and SpO_2_ shown on the desktop screen at the central nurses’ station. Figure 3 suggests that nurses returned to observe patients for whom they had a concern within 120 minutes during unmonitored periods but did not do so during monitored periods, suggesting that wearable monitoring may have provided reassurance that the patients’ condition was unchanged. This reduced entries to the isolation rooms, minimizing risks of exposure to the virus and saving nurse time in donning and doffing PPE.

#### Impact on Patient Safety

Although our data show that the TTNO after an EWS of 3 or more was mostly longer than the protocolized 60 minutes (median of 123 and 139 minutes without and with wearables, respectively), this delay in hourly observation measurements is recognized as a common feature in both pandemic and nonpandemic conditions [22,23], with compliance as low as 30% [24]. Multiple barriers have been identified that contribute to these delays, which are likely to have been worsened in the context of the pandemic [25].

There was no difference in the EWS distributions (*P*=.92; Figure 4) for the times with wearable monitoring and those without it evaluated on the fourth quartile of TTNO distribution as this represents the longest intervals between consecutive observations. Figure 3 shows that, proportionally, there were many more instances of increased TTNOs during the monitored periods (WMS in use) with respect to the unmonitored periods (no WMS). Figure 4 provides some reassurance that this difference was not associated with increased physiological instability (increase in EWS with respect to the previous observation). However, this finding should be considered in the context of our exploratory observational study design. This was also identified in our parallel qualitative study of staff use of the system [26]. Interviews with multi-professional staff demonstrated that the WMS afforded reassurance that the patient was stable between vital sign observations, allowing nurses to manage their workload as they felt appropriate. Although this perceived prioritization was reflected in the exploratory TTNO analyses, with larger TTNOs during stable periods than during unstable periods (+51 vs +16 minutes; *P*<.001 for both; Table 2), adjusted analyses did not demonstrate a significant difference between stable and unstable periods.

Similar to other work [27], we have shown that episodes of moderate and severe vital sign deviations in hospitalized patients with COVID-19 are common. Without continuous monitoring, detection of deterioration may be delayed until the next set of nurse observations as no additional vital sign data are available. Continuous SpO_2_ monitoring can be considered an easily applicable tool for the early recognition of patients with the potential for acute respiratory deterioration. The wearable data values and associated EWSs were updated every 2 seconds on the desktop screen (for multiple patients in parallel in the real-time dashboard view), and their trends, updated every 5 minutes using the vital sign medians, were stored and could be reviewed at any time (patient chart view). The lack of visual and audible alerts, apart from the EWS traffic light color scheme for its display on the desktop screens, did not allow us to know whether the nurses were observing the screen when deterioration set in.

Our finding that, during monitored periods, TTNO could be extended suggests that wearables may help nurses with clinical time management. Any changes resulting from the implementation of a WMS would also need to be reflected in the approach to vital signs measurements in standard care, for example, in the frequency of observations for stable patients.

Several barriers to the successful in-hospital deployment of clinical-grade wearable technologies on a larger scale are still to be overcome, including cost, reliability, efficiency, and the use of data processing systems [3,5,28]. In future work, the use of the National Early Warning Score 2 scoring system, adopted nationwide in the United Kingdom from April 2021, after the data reported in this paper were acquired, would provide more generalizable results, with a broader understanding and easier applicability.

### Strengths and Limitations

The prospective registration and the use of a rigorously developed unique WMS are strengths of this study. However, there are limitations that should be considered. Although we report a novel analysis that has the potential to advance our understanding of the impact of WMSs on nurse behavior, this analysis was post hoc and not included in our study registration.

The WMS was deployed under considerable pressure in the context of the COVID-19 pandemic and into a dynamic clinical setting including 3 different wards, which incurred several limitations. First, nurse behaviors during this time may have varied depending on the ward setting or evolved during the course of the pandemic and may not be comparable to conditions since the pandemic given the very high workload and stress at that time. Second, due to limited availability of equipment, nursing staff chose which patients to monitor using the system, which may have introduced selection bias as they were more likely to monitor those assessed at highest risk of deterioration. In addition, nurses may have taken fewer intermittent vital sign observations as patients recovered and neared discharge. However, discharge from hospital was accelerated during the pandemic due to resource pressures, which may have offered some mitigation against this potential confounder of the TTNO analyses. Third, the comparison between different time windows for the same patients could have been influenced by clinical staff who knew the patients by the time they were on the WMS. Thus, the observed pattern of monitoring might be more (or less) spaced given this potential bias and reflect the impact of competing clinical priorities. Fourth, the criteria for hospital admission and discharge evolved across the 2 pandemic waves, resulting in variability between patient clinical acuity at hospital admission and discharge, potentially confounding the TTNO analyses. Given the nature of this study, it was not possible to compare a completely nonmonitored cohort, which would have mitigated these first 4 limitations. Future research under nonpandemic conditions should consider using a separate unmonitored control group and setting specific eligibility criteria for commencing and ceasing monitoring to facilitate consistency of use. Fifth, although vital sign values from nurse-measured and wearable sources were compared, these 2 methods of measurement do incur differences, such as accuracy of devices and mode of measurement, which should be considered in the context of this analysis. In particular, nurse-measured respiratory rates may differ significantly from those measured using the WMS due to monitoring behaviors such as “digit preference” (where respiratory rate distribution shows peaks at 20 and 24 breaths per second). This suggests either estimation of respiratory rate based on observation or measuring over a shorter period of 15 seconds and multiplying by 4 [29,30]. Future research should consider protocolizing nurse-measured vital signs to ensure accuracy and avoid these behaviors.

We identified one limitation related to the WMS, which included a pulse oximeter device known to consistently underestimate oxygen saturation by up to 2%. However, the device was selected as the most reliable and accurate of those tested for the system [9], and notification of this underestimation was included in staff training and deemed by the nurses to be manageable and preferable to overestimation [26]. This may have resulted in nurses unnecessarily returning to patients to check their vital signs but did not provide false reassurance or risk masking hypoxia. Future studies should consider the impact of the comparative accuracy of devices on nurse monitoring behaviors.

There are some limitations to the data available. First, we planned to include additional adverse outcomes, including escalation to high-frequency nasal oxygen and noninvasive ventilation, in our analysis. However, these data were not available in the extracted dataset as patients undergoing these interventions were moved to a separate high-dependency ward. Future studies should ensure that data on these additional outcomes are available. Second, 5.7% (9/157) of the initial patients were not included in the analysis as they had registered for the NHS digital data opt-out service. Although this omission may pose a risk of selection bias, given the small number, this is unlikely to have impacted the results. Future research using electronic patient data will need to consider how missing data due to the opt-out service are handled if the proportion of patients choosing to opt out increases over time.

From a modeling perspective, TTNO was right skewed. Although we used a linear mixed effects model to preserve the interpretability of effect estimates as absolute differences in minutes, sensitivity analyses using alternative distributional assumptions (gamma and log-normal models) provided consistent results. However, departures from normality may have affected estimate precision.

Finally, our data were from a single hospital with a high percentage of ICU admissions (31/144, 21.5%). We acknowledge that these factors limit the generalizability of our findings. Future research should focus on exploring the impact of WMSs on nurse behaviors in nonpandemic conditions and comparing robust and distinct patient groups. Future consideration should also be given to the cost-benefit of the system given the impact on nurse behaviors and patient safety.

### Conclusions

In this retrospective data study, patients were at high risk of deterioration, spending a third of the monitored time at or above the escalation threshold. Our post hoc analysis showed that, by offering additional vital sign data between intermittent EWS measurements, the WMS may have improved task prioritization by allowing the nursing staff to extend the time between routine MVSMs. We found no indication in our analysis that this increase in time between vital signs measurements adversely affected patient safety. The increase in time between observations was not associated with a difference in EWS distributions, providing some reassurance that this difference was not associated with increased physiological instability.

## Appendix

Multimedia Appendix 1 Cohort age distribution and levels of agreement between wearable monitoring system devices and the nurse intermittent manual vital sign measurements.

Multimedia Appendix 2 Graphs presenting physiological patterns of vital signs.

## Abbreviations

EPR: electronic patient record
EWS: early warning score
ICU: intensive care unit
MVSM: manual vital sign measurement
NHS: National Health Service
PPE: personal protective equipment
SpO2: peripheral oxygen saturation
TTNO: time to next observation
WMS: wearable monitoring system

## Data Availability

The datasets generated or analyzed during this study are available from the corresponding author on reasonable request.
