# Correction: Clinical Evaluation of a Real-Time Wearable System for Monitoring In-Hospital Ambulatory Patients With COVID-19: Retrospective Data Study

**DOI:** 10.2196/109596

**Published:** 2026-10-22

**Authors:** Sarah Vollam, Cristian Roman, Mauro Santos, Marco Pimentel, Oliver Redfern, Lionel Tarassenko, Peter Watkinson

**Affiliations:** 1 Nuffield Department of Clinical Neurosciences University of Oxford Oxford, England United Kingdom; 2 NIHR Oxford Biomedical Research Centre Oxford United Kingdom; 3 Institute of Biomedical Engineering, Department of Engineering Science University of Oxford Oxford United Kingdom; 4 Oxford Critical Care John Radcliffe Hospital Oxford United Kingdom

In “Clinical Evaluation of a Real-Time Wearable System for Monitoring In-Hospital Ambulatory Patients With COVID-19: Retrospective Data Study” [1], the authors have requested that the two authors listed first—SV and CR—be recognized as joint first authors with equal contribution. This was indicated in the original submitted metadata but removed during publisher processing.

The metadata has been amended to include an asterisk after both SV’s and CR’s names, indicating equal contributions.

The author list has been revised from the following:

Sarah Vollam^1,2^, PhD; Cristian Roman^2,3^, PhD; Mauro Santos^3^, DPhil; Marco Pimentel^3^, DPhil; Oliver Redfern^1^, PhD; Lionel Tarassenko^2,3^, DPhil; Peter Watkinson^1,2,4^, MD

The author list now reads:

Sarah Vollam^1,2*^, PhD; Cristian Roman^2,3*^, PhD; Mauro Santos^3^, DPhil; Marco Pimentel^3^, DPhil; Oliver Redfern^1^, PhD; Lionel Tarassenko^2,3^, DPhil; Peter Watkinson^1,2,4^, MD

*these authors contributed equally

The correction will appear in the online version of the paper on the JMIR Publications website together with the publication of this correction notice. Because this was made after submission to PubMed, PubMed Central, and other full-text repositories, the corrected article has also been resubmitted to those repositories.

## References

[1] Vollam Sarah, Roman Cristian, Santos Mauro, Pimentel Marco, Redfern Oliver, Tarassenko Lionel, Watkinson Peter (2026). Clinical evaluation of a real-time wearable system for monitoring in-hospital ambulatory patients with COVID-19: retrospective data study. JMIR Med Inform.

