# Supplementary material for: Clinical Evaluation of a Real-Time Wearable System for Monitoring In-Hospital Ambulatory Patients With COVID-19: Retrospective Data Study
Source: JMIR Med Inform. 2026 Jun 22;14:e81304. doi: 10.2196/81304 (PMC13338678; doi:10.2196/81304)
Supplement: Multimedia Appendix 1 [file medinform_v14i1e81304_app1.docx]

**Clinical evaluation of a real-time wearable system for monitoring in-hospital ambulatory COVID-19 patients: A retrospective data study**

**MULTIMEDIA APPENDIX A:**

**ADDITIONAL TABLES**

Table AA1. Types and sources of vital-signs data recorded. MVSM – manual vital sign measurements, recorded into the hospital electronic system by clinical staff.

| **Vital Sign** | **Wearables** | **MVSM** |
| --- | --- | --- |
| **Heart Rate (HR)** | VitalPatch | Not measured |
| **Pulse Rate (PR)** | Nonin 3150 Ox_2_ | GE Healthcare Dinamap monitor |
| **Respiratory Rate (RR)** | VitalPatch | Clinical Visual Assessment |
| **Oxygen Saturation (SpO_2_)** | Nonin 3150 Ox_2_ | GE Healthcare Dinamap monitor |

Levels of agreement between vital-signs recorded by the wearables and the nurse intermittent MVSM (wearables minus MVSM)


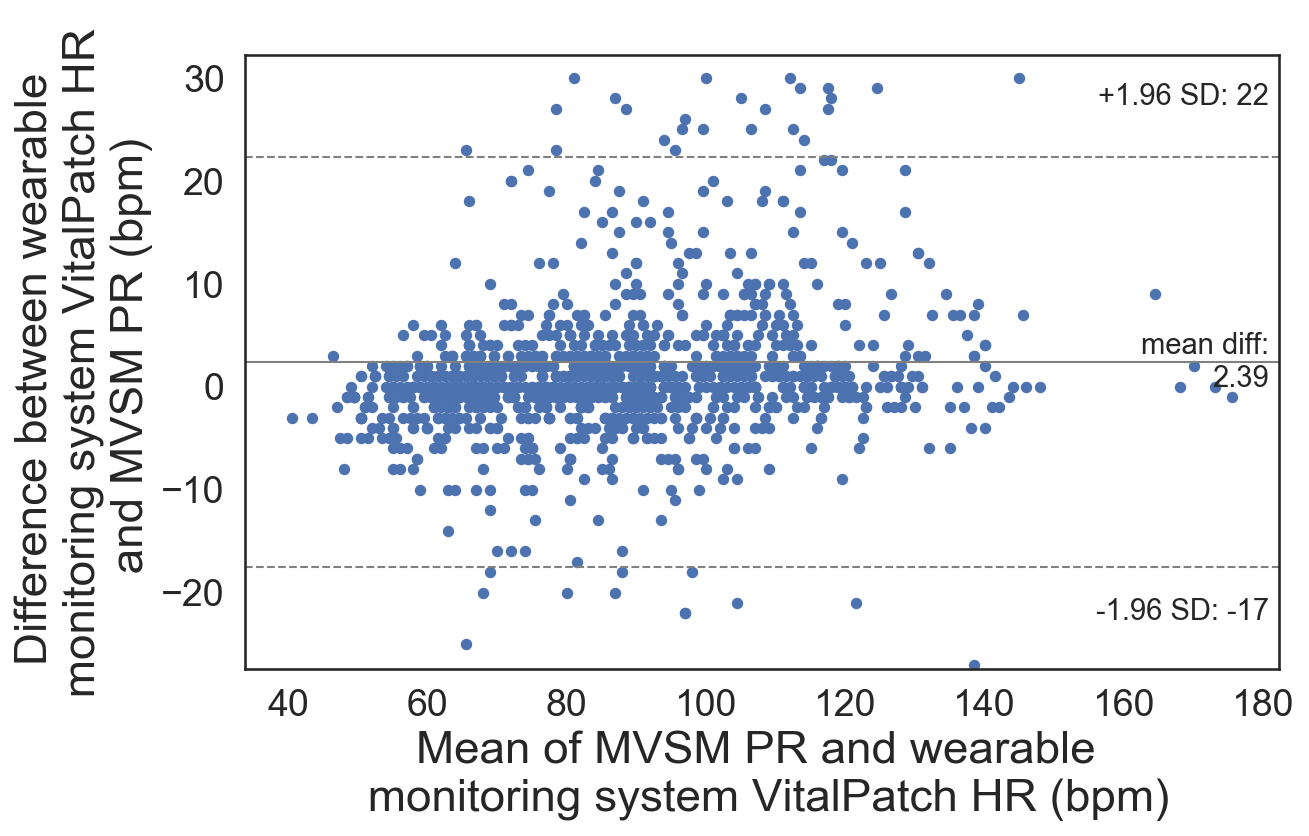
Figure AA1. Bland-Altman plot for all available MVSM for Pulse Rate vs wearable VitalPatch Heart Rate estimates, computed as the median value within the 5-minute window adjacent to the MVSM value– in beats per minute (bpm). The mean difference is +2.39, CI [-17, 22] bpm.


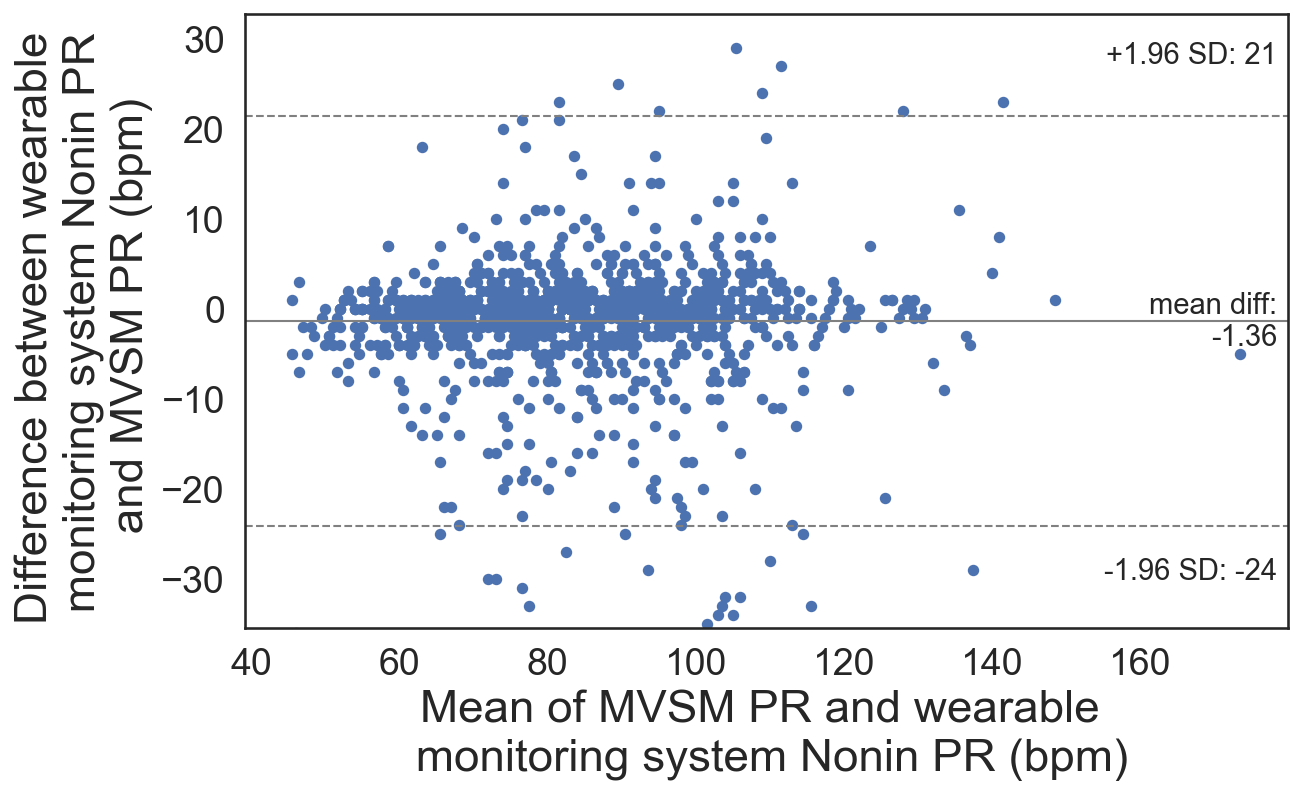


Figure AA2. Bland-Altman plot for all available MVSM for Pulse Rate vs wearable Nonin Pulse Rate estimates, computed as the median value within the 5-minute window adjacent to the MVSM value– in beats per minute (bpm). The mean difference is -1.36, CI [-24, 21] bpm.


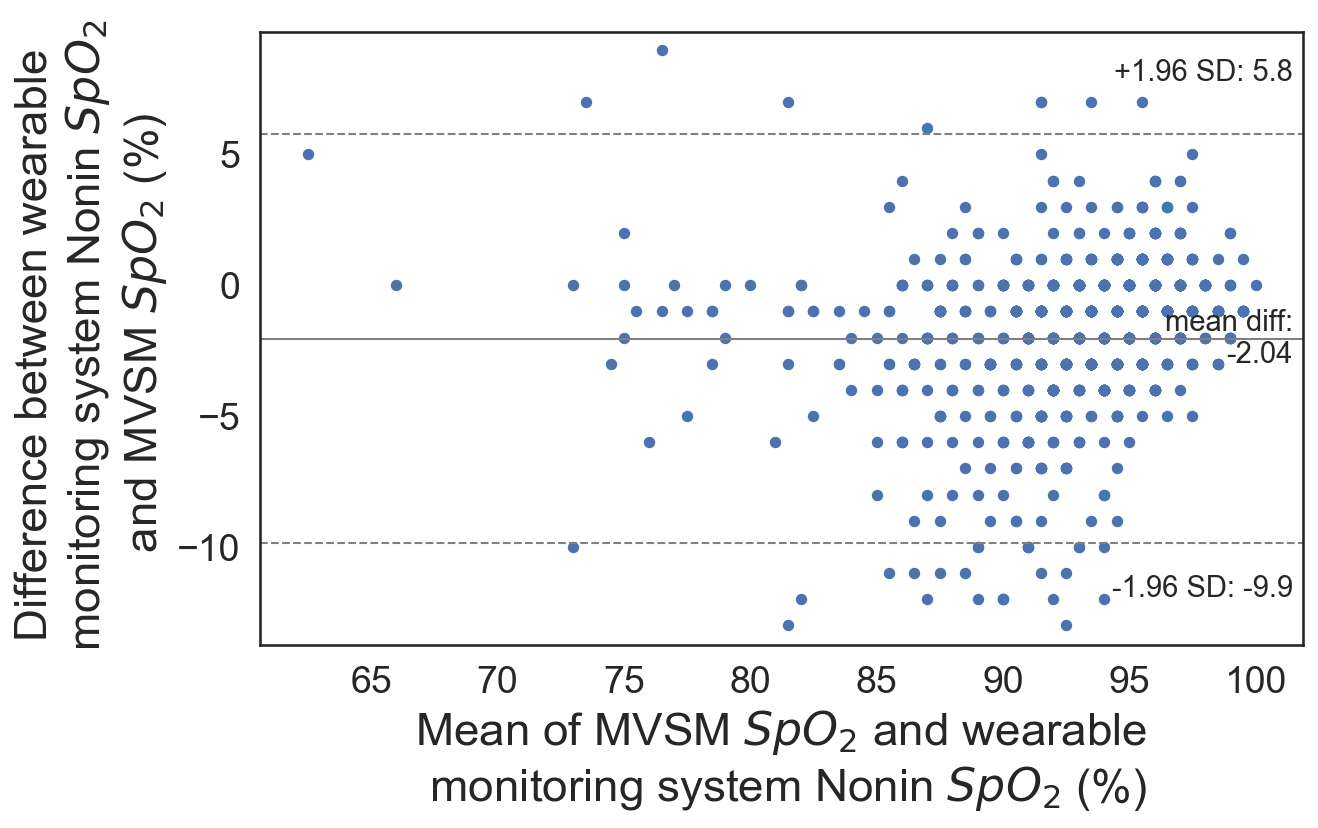


Figure AA3. Bland-Altman plot for all available MVSM for SpO_2_ vs wearable Nonin SpO_2_ estimates, computed as the median value within the 5-minute window adjacent to the MVSM value – in percentage (%). The mean difference is -2.04, CI [-9.9, 5.8] %.


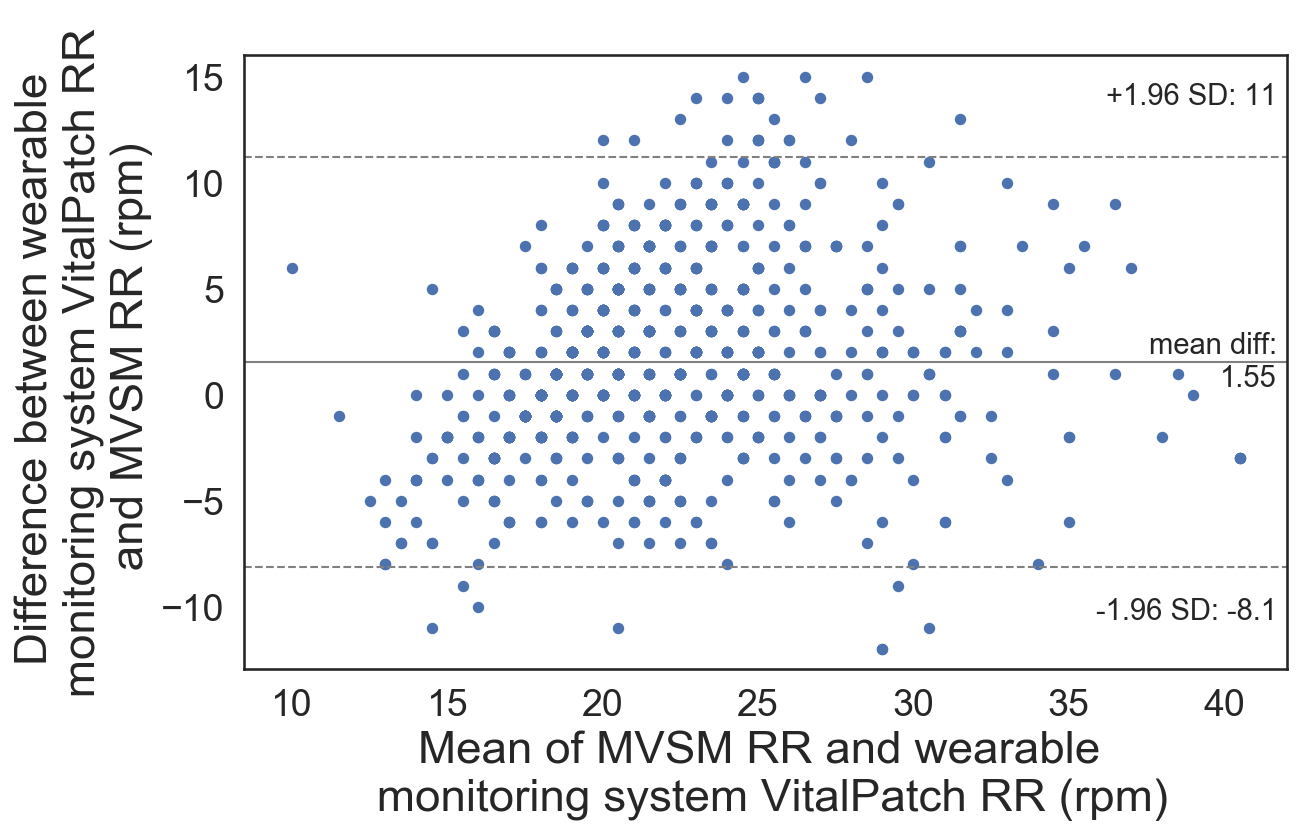


Figure AA4. Bland-Altman plot for all available MVSM for Respiratory Rate vs wearable VitalPatch Respiratory Rate estimates, computed as the median value within the 5-minute window adjacent to the MVSMvalue – in respirations per minute (rpm). The mean difference is +1.55, CI [-8.1, 11] rpm.


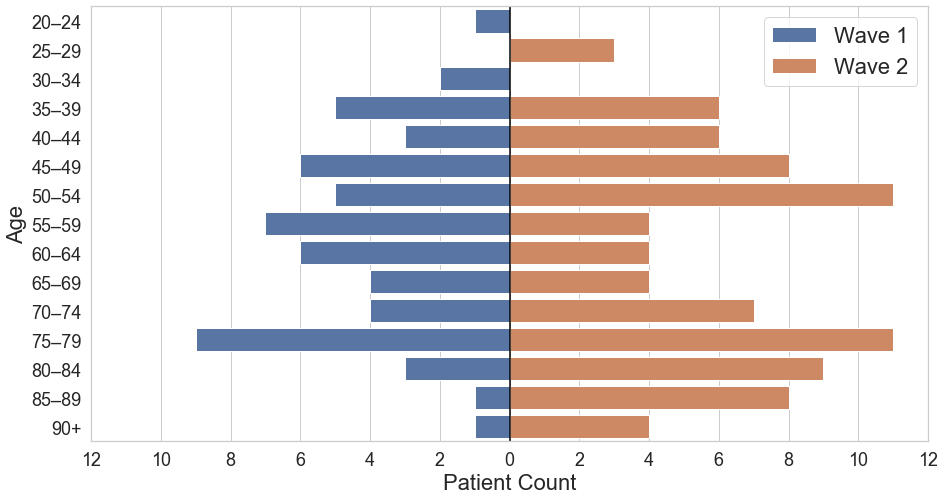


Figure AA5. Age distribution (years) of patients admitted in Wave 1 (March to August 2020) (n = 57) and in Wave 2 (September 2020 – February 2021) (n = 87)
