# Supplementary material for: Clinical Evaluation of a Real-Time Wearable System for Monitoring In-Hospital Ambulatory Patients With COVID-19: Retrospective Data Study
Source: JMIR Med Inform. 2026 Jun 22;14:e81304. doi: 10.2196/81304 (PMC13338678; doi:10.2196/81304)

**Clinical evaluation of a real-time wearable system for monitoring in-hospital ambulatory COVID-19 patients: A retrospective data study**

**MULTIMEDIA APPENDIX 2:**

**GRAPHS PRESENTING PHYSIOLOGICAL PATTERNS OF VITAL SIGNS**

In this supplementary appendix we present additional data on physiological patterns of vital signs from continuous monitoring for hospitalised patients treated for COVID-19 infection outside of critical care. Trajectory figure AB1 displays wearable data indexed to relative hospital time, with monitoring plotted only during the proportion of admission in which it occurred. Figures AB2 and AB3 display wearable data relative to the WMS monitoring time. Although trajectories indexed to relative WMS monitoring time should be interpreted with caution given variability in monitoring duration, this representation facilitates comparison across categories by aligning patients to comparable phases of wearable monitoring. In this case, relative time is useful to assess staff workflow: whether wearable monitoring is initiated during patient instability, whether patients improve during wearable monitoring, or whether monitoring is stopped after a patient is more stable. Figures AB2 and AB3 should not be used to model deterioration relative to admission. All figures display 95% confidence intervals to illustrate the precision of estimated trajectories.

In trajectory example 1 (figure AB1) there is a clear distinction between those who died compared with those who survived. For those patients who died, deterioration appeared to begin 60% of the way through their hospital stay. In trajectory example 2 (Figure AB2) those who died exhibited consistently a higher EWS score (approx. +1) throughout the duration of monitoring than those who survived. Patients admitted during wave one demonstrated higher overall EWS trajectories than those admitted during wave two (Figure AB3). Figures AB2 and AB3 reveal broadly similar trajectory patterns within monitoring categories consistent with relatively stable criteria for system use in practice.

**Figure AB1: trajectory example 1.** Trajectory of WMS-recorded EWS for patients relative to their hospital length of stay, including 95% confidence intervals.


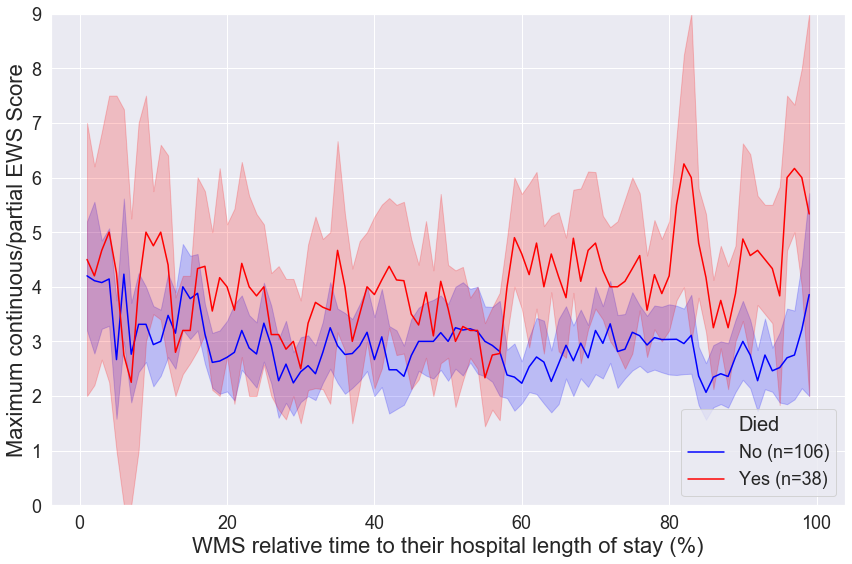


**Figure AB2: trajectory example 2.** WMS-recorded EWS trajectory for patients that ***died*** vs those that did ***not*** relative to their WMS monitoring duration, including 95% confidence intervals.


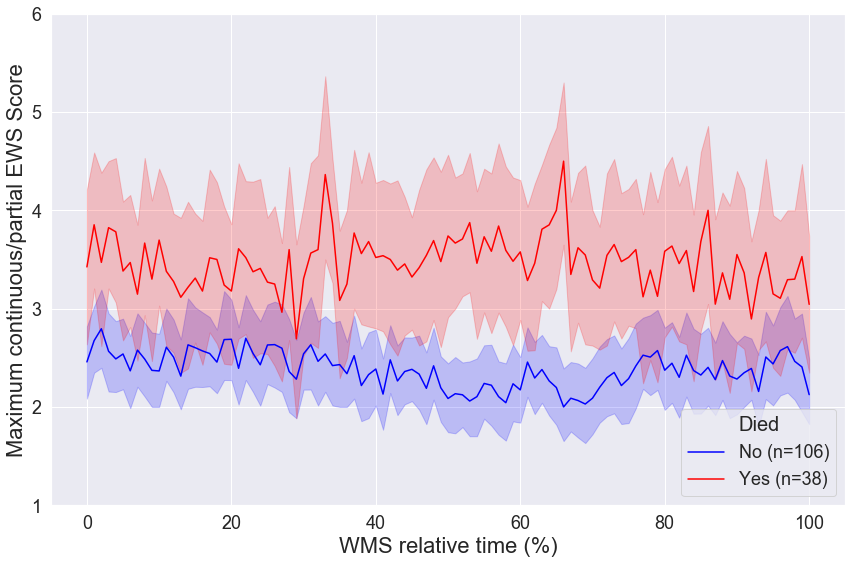


**Figure AB3: trajectory example 3.** WMS-recorded EWS trajectory for all patients, relative to their WMS monitoring duration, split into wave 1 (March to August 2020) and wave 2 (September 2020 – February 2021) of the pandemic, including 95% confidence intervals.


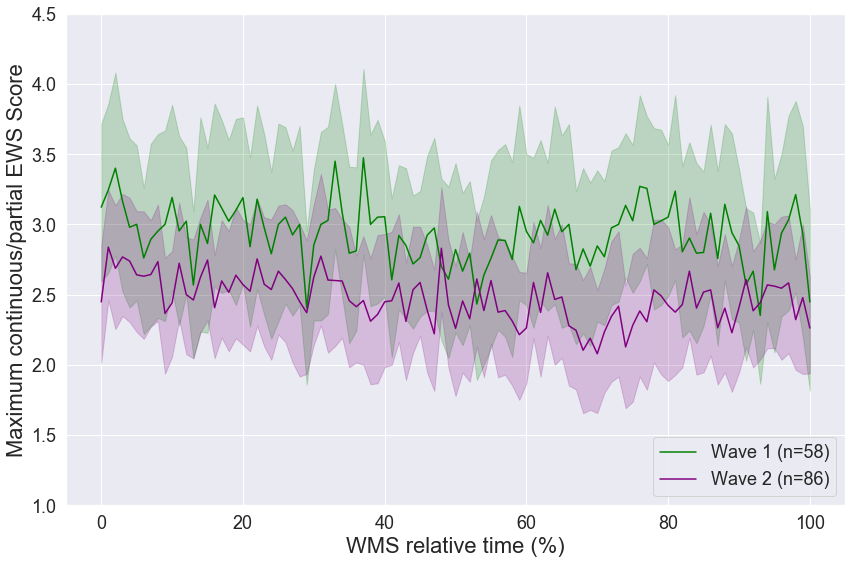

Supplement: Multimedia Appendix 2 [file medinform_v14i1e81304_app2.docx]
